# Intragenic tandem repeats in *Daphnia magna*: structure, function and distribution

**DOI:** 10.1186/1756-0500-2-206

**Published:** 2009-10-06

**Authors:** Isabelle Colson, Louis Du Pasquier, Dieter Ebert

**Affiliations:** 1 Basel University, Zoological Institute, Vesalgasse 1, CH-4051 Basel, Switzerland

## Abstract

**Background:**

Expressed sequence tag (EST) databases provide a valuable source of genetic data in organisms whose genome sequence information is not yet compiled. We used a published EST database for the waterflea *Daphnia magna *(Crustacea:Cladocera) to isolate variable number of tandem repeat (VNTR) markers for linkage mapping, Quantitative Trait Loci (QTL), and functional studies.

**Findings:**

Seventy-four polymorphic markers were isolated and characterised. Analyses of repeat structure, putative gene function and polymorphism indicated that intragenic tandem repeats are not distributed randomly in the mRNA sequences; instead, dinucleotides are more frequent in non-coding regions, whereas trinucleotides (and longer motifs involving multiple-of-three nucleotide repeats) are preferentially situated in coding regions. We also observed differential distribution of repeat motifs across putative genetic functions. This indicates differential selective constraints and possible functional significance of VNTR polymorphism in at least some genes.

**Conclusion:**

Databases of VNTR markers situated in genes whose putative function can be inferred from homology searches will be a valuable resource for the genetic study of functional variation and selection.

## Background

Waterfleas of the genus *Daphnia *(Crustacea:Cladocera) are small planktonic crustaceans found in standing freshwater bodies around the world. They have a long history as model organisms for evolutionary, ecological and ecotoxicological research. Recently, the genus has been the focus of a major sequencing effort, and the full genome sequence of *Daphnia pulex *is now available [1]. Genomic resources are steadily being developed for another species of the genus, *D. magna*. In particular, a database of around 12,000 expressed sequence tags (EST) is currently available [1,2], providing a useful resource to isolate polymorphic genetic markers in this species. Developing genetic markers from transcribed sequences offers specific advantages compared to traditional methods of screening enriched genomic libraries. Apart from the lower cost and higher speed of development, EST-derived genetic markers have a higher probability of being functionally significant and of being located in gene-rich regions [3-5]. This makes them highly useful markers for QTL mapping of ecologically-relevant phenotypes and for the study of selection in natural populations. Although it could be thought that functional constraints might limit polymorphism levels in genic repeated sequences, comparative studies have reported both lower [6] and higher [4] levels of polymorphism in genic microsatellites as compared with genomic microsatellites. Polymorphism of transcribed repeated sequences can have direct phenotypic consequences both in terms of protein function [7] and in terms of regulating gene expression [8]; it has also been hypothesised to play an important role in evolvability and phenotypic adaptation [9-12]. Here, we report the development of 74 polymorphic VNTR markers from the *D. magna *EST database and explore their patterns of polymorphism in relation to repeat sequence structure and putative gene function.

## Methods

The Tandem Repeat Finder (TRF) software [13] was used to recover tandemly repeated sequences from the *D. magna *EST database [1]. Sequences containing only mononucleotides repeats were discarded, and redundant sequences were merged using the CAP assembly software [14]. The 346 single sequences obtained from 531 ESTs were translated using the expasy "Translate" software [15]. The amino acid sequence of the longest open reading frame (ORF) was then blasted against protein databases [16] and used in InterProScan searches [17] in order to identify functional domains and transmembrane regions. E-values < 0.0001 were accepted as significant homology in the blast searches. When translation did not produce an obvious candidate ORF, blastX searches were carried out from the nucleotide sequence. Putative function was inferred from the identity of homologous sequences and from the presence of functional domains. Six broad functional categories were defined: 1. Proteins involved in metabolism, including energy metabolism and protein synthesis (MET); 2. Proteins involved in signalling pathways and regulation of gene expression (SIG); 3. Surface or integumental proteins (SUR); 4. Proteins involved in defense (pathogens and stress) (DEF); 5. Other proteins with known function (OTH), regrouping proteins involved in development, transport and cell structure, functions that were represented by only a few loci; 6. Proteins of unknown function (UNK), regrouping loci with non-annotated homologous sequences and loci with no significant homologous sequence in Genbank. The position of the tandem repeat in the mRNA sequence (ORF, 5'UTR or 3'UTR) was determined using the gene prediction software FGenesh [18,19]. Primers were designed for 218 loci, using the "Primer 3" software [20]. DNA from 18 *D. magna *individuals representing six populations from Europe and North-America (UK, Germany, Belgium, Finland, Hungary and Canada) was extracted with E.Z.N.A tissue DNA mini kit (Peqlab, Germany) and used in PCR reactions. Depending on the locus, we performed either standard or hot start PCR. Standard PCR reactions were carried out in 12.5 μl reactions containing 1× PCR reaction buffer (Sigma Aldrich), 1.5 or 3.5 mM MgCl_2 _depending on the locus, 200 μM of each dNTP, 0.2 μM of each primer (with the forward primer fluorescently labelled) and 0.5 unit Taq polymerase (Sigma Aldrich). An initial denaturation step of 4 minutes at 94°C was followed by 35 cycles of 94°C for 30 seconds, 53°C for 30 seconds, and 72°C for 30 seconds, followed by a final extension step of 72°C for 4 minutes. Hotstart PCR was performed with thermo-start PCR master mix (ABGene, Epsom, UK) with 1.5 mM or 3.5 mM of MgCl_2 _depending on the locus, and 0.2 μM of each primer (with the forward primer fluorescently labelled). PCR conditions were as described above, except for an initial incubation at 94°C for 15 minutes. Primer sequences and PCR conditions for polymorphic VNTR loci are described in Additional File 1. PCR products were run on an ABI 310 automated sequencer (Applied Biosystems, Foster City, USA) and analysed with the Genemapper software (Applied Biosystems, Foster City, USA). Polymorphism (number of alleles) was assessed at 106 loci, which consistently amplified DNA from all individuals and for which no more than 2 alleles per individual were present. Furthermore, the 106 loci were blasted against the *D. pulex *genome to check for any potential gene duplication.

We analysed contingency tables using the χ^2 ^test when sample sizes were large enough (less than 20% of cells containing less than 5 cases). Otherwise, Yates' correction was employed [21]. We conducted nonparametric correlations using Spearman rank correlation factor rho, performed with SPSS 15.0.

## Results and Discussion

**Figure 1:**
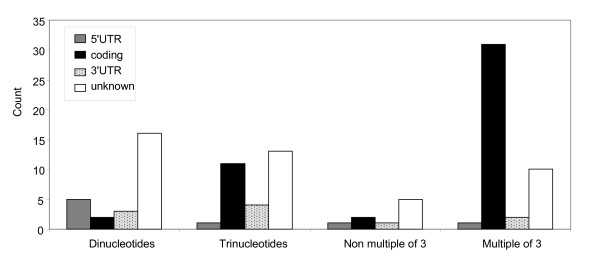
**Distribution of repeat structures and localisation in the mRNA**. Localisation of repeated motifs predicted with the FGenesh software. "5'UTR": 5' untranslated region; "coding": protein coding region; 3'UTR: 3' untranslated region; "unknown": loci for which the location of the repeated motif could not be ascertained. Total number of loci = 106.

Most repeated sequences contained trinucleotide repeats (29), or longer repeats with a number of nucleotides (44) divisible by 3, hereafter referred to as "in-frame" repeat sequences (Additional file 2). This is to be expected in coding regions, as polymorphism in the number of repeats will not lead to frameshift mutations. As expected, most in-frame repeated sequences were situated in the coding region of the mRNA (Figure 1, Additional files 1 and 2) (42 located in coding region, 2 located in 5'UTR, 6 located in 3'UTR, for 23 of unknown location). Dinucleotide repeats seem to be preferentially located in the untranslated regions of the mRNA (5 in 5'UTR, 3 in 3'UTR and 2 in coding region). However, the position of repeats could not be characterised within the majority of dinucleotide repeat sequences (16). Finally, a small number (9) of longer off-frame repeats (with a repeat size not divisible by three) were also observed. Preferential location of in-frame repeats in the coding region and off-frame repeats in the untranslated region were statistically significant (Fisher Exact Test, p < 0.001).

**Figure 2:**
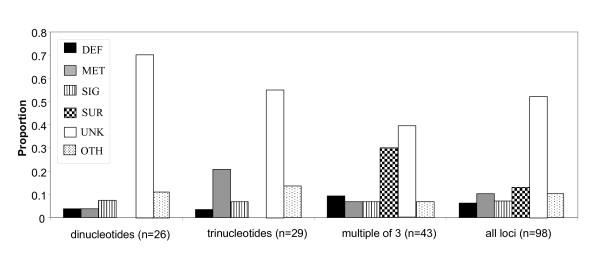
**Functional distribution of EST loci containing tandem repeats**. Longer "off-frame" repeats were not included in the analysis due to their low number. Total number of loci = 98.

Forty-six percent of loci could be assigned a putative function (Additional File 3). Metabolism genes were slightly over-represented in the trinucleotide repeats (accounting for 21% of loci containing trinucleotide repeats, compared to 10% of all loci), while surface and integumental protein genes were over-represented in ESTs with in-frame repeat motifs of more than 3 nucleotides (accounting for 30% of these loci, compared to 12% of all loci). All identified surface and integumental proteins harboured arrays of long in-frame repeats (Figure 2). These integumental proteins were mainly composed of cuticular chitin-binding proteins (12 out of 13).

Out of 106 loci tested, 74 (70%) were polymorphic across the six tested populations, although not necessarily within each population. However, the small number of genotyped individuals did not allow for a meaningful estimation of population-level polymorphism (see Additional File 4). Our data suggest that different levels of diversity exist in different geographical locations, with the lowest diversity observed in Canada. Further studies will be needed to determine the relationship of these differences to life history, population history or natural selection variables.

The proportion of polymorphic loci was independent of repeat structure (χ^2 ^= 3.048, df = 3, p > 0.05), repeat localisation in mRNA (χ^2 ^= 0.87, df = 2, p > 0.05, with Yates correction), and putative protein function (χ^2 ^= 1.88, df = 4, p > 0.05, with Yates correction). However, putative defense genes showed a higher proportion of polymorphic loci (5 out of 6) than other functional categories. The number of alleles was significantly positively correlated with the number of repeats (Spearman rank correlation coefficient: 0.303, p < 0.01 between total number of repeats and number of alleles; 0.218, p < 0.05 between number of perfect repeats and number of alleles).

Homology searches against the *D. pulex *genome identified 49 EST with partial homology of fragments longer than 100 base pairs. Eleven of these had multiple partial homologues situated in distinct genomic locations in the *D. pulex *genome, indicating some degree of sequence duplication and paralogy in the *D. pulex *genome. However, most of the homologue sequences (39) were only partial and not encompassing the whole amplicon (with either one or both primer sequences missing). We found that only ten loci had a *D. pulex *homolog encompassing the whole amplicon, i.e. including both primer sequences in the same scaffold, allowing for the presence of introns. In all cases, only one complete homologue was identified. From this analysis and in view of the genotyping results we are confident that, although gene duplication seems to be a common feature in the *Daphnia *genome (at least in *D. pulex*), our genetic markers represent single loci.

The EST database allowed fast, cheap *in silico *screening for potential VNTR genetic markers in *Daphnia magna*. From 346 single sequences identified, primers could be designed for 218 loci. For 106 loci, it was possibly to amplify the DNA of all individuals from six distinct locations. The majority of repeat motifs showed "in-frame" polymorphism. These repeat motifs are preferentially located in coding regions and non-randomly distributed among putative gene functions: trinucleotides are preferentially found in genes linked to metabolism and intracellular processes, while longer in-frame repeat motifs (9 to 39 bp) are present in surface or integumental proteins, essentially cuticular proteins. In-frame polymorphic repeats have been shown to be functionally important [7], in particular in integumental proteins. Cuticular proteins often contain a hydrophobic tetrapeptide repeat, which could be involved in the exoskeleton mechanical characteristics [22]. In *Saccharomyces cerevisiae*, most genes containing intragenic repeats encode cell-wall proteins, and variability in the number of repeats has been linked to variability in adhesion properties [7]. This indicates that polymorphism in cuticular protein VNTR markers might be functionally significant for both exoskeleton structure and in the defense against pathogens, as many cuticular proteins possess antimicrobial properties [23], and natural populations of *D. magna *are known to harbour many parasites [24,25].

Seventy-four loci were found to be polymorphic, with the number of alleles ranging from 2 to 10. The proportion of polymorphic loci was independent of repeat structure, location of the repeat in the mRNA, protein function and cellular localisation of protein product. However, most defense-related genes (5 out of 6) were polymorphic, with a relatively high number of alleles (4 to 8). Similarly, there was a non significant trend for loci coding of surface and extracellular proteins to have a higher proportion of polymorphic loci (28/39, 72%) than loci coding for intracellular proteins (17/31, 55%). These trends can tentatively be interpreted as repeated sequences playing a role in the evolutionary dynamics of host-pathogen relationships (see [7] for a discussion of this topic in pathogens). However, more data and much more targeted analyses, which fall outside the scope of this report, are needed to further explore this possibility.

We observed a significant positive correlation between polymorphism and number of repeats amongst our loci, as previously observed in genomic microsatellites [26]. However, interruption of the length of perfect repeat array did not correlate with lower polymorphism, as is the case in genomic microsatellites [27]. This discrepancy could be explained by differences in mutational and selective constraints in intragenic and genomic microsatellites, in particular in relation to third codon position redundancy. Also, our dataset includes loci with longer repeat structures ("minisatellites") for which replication slippage might not be the primary mutational process.

EST databases are increasingly being used as a resource to develop VNTR markers, which are likely to be very informative in genome screens for functionally relevant polymorphism. The 74 VNTR markers for *D. magna *described here will be useful in producing the first genetic linkage map in the species (increasing marker density in gene rich areas), and in QTL mapping of evolutionary and ecologically relevant traits. To illustrate the potential of our VNTR markers, 34 of the 74 polymorphic markers described here were found to distinguish between two European clones used to develop recombinant lines for mapping purposes (unpublished data). The availability of markers with potentially functionally relevant polymorphism, coupled with information on the putative function of the gene product, can also help researchers target candidate markers possibly linked with phenotypes of interest.

## Competing interests

The authors declare that they have no competing interests.

## Authors' contributions

IC and DE participated in the design of the study. IC carried out the in silico marker development and functional searches, most of the genotyping and data analysis, and drafted the manuscript. LDP participated in the design of the functional analysis and critically revised the manuscript. DE conceived of the study and critically revised the manuscript. All authors read and approved the final manuscript

## Supplementary Material

Additional file 1 **PCR conditions for polymorphic VNTR loci**. Label: F-primer fluorescent label used. [MgCl_2_]: Concentration of MgCl_2 _used in the PCR buffer. An asterisk following MgCl_2 _concentration indicates hot start PCR. Click here for file

Additional file 2 **Description of the 74 polymorphic VNTR loci**. EST locus: locus containing the VNTR; Size: size of the repeated motif; Sequence: repeat consensus sequence. N: number of repeats, [the number of perfect repeatsm with exact consensus sequence, is shown in brackets]; A: number of alleles. *: total number of alleles in loci with more than one repeated motif. He: Gene diversity. Click here for file

Additional file 3 **Results of homology searches and putative functions of EST loci**. TM: transmembrane regions. Function categories: DEF: defense; MET: metabolism; OTH: other; SIG: signaling and gene expression regulation; SUR: surface and integumental proteins; UNK: unknown function. No hit: no significant homolog found (cut-off E-value 0.0001). Click here for file

Additional file 4 Distribution of allele sizes of polymorphic loci in each sampled location. Click here for file
