# Supplementary material for: Intragenic tandem repeats in Daphnia magna: structure, function and distribution
Source: BMC Res Notes. 2009 Oct 6;2:206. doi: 10.1186/1756-0500-2-206 (PMC2763877; doi:10.1186/1756-0500-2-206)
Supplement: Additional file 1 — PCR conditions for polymorphic VNTR loci. Label: F-primer fluorescent label used. [MgCl2]: Concentration of MgCl2 used in the PCR buffer. An asterisk following MgCl2 concentration indicates hot start PCR. [file 1756-0500-2-206-S1.DOC]

**Additional file 1**. PCR conditions of polymorphic VNTR loci. Label: F-primer fluorescent label used. [MgCl_2_]: Concentration of MgCl_2_ used in the PCR buffer. An asterisk following MgCl_2_ concentration indicates hot start PCR.

| **ESTlocus** | **Forward primer** | **Reverse primer** | **Label** | **[MgCl_2_]** |
| --- | --- | --- | --- | --- |
| WFes0001245 | TTATCGTAGCGCTGATGCTC | TGAGCTCGCCGAAAAGTTAT | 6-FAM | 1.5 |
| WFes0001508 | TGCTTTGCCATAAGGAGACA | AACTGCCTCCCCAACTTTTT | TET | 1.5 |
| WFes0001526 | AGCTAGCGCCATCTGTTCAC | ACAGATTGAAGCGAGGCATT | HEX | 1.5 |
| WFes0001668 | CTGGACTGTGTGCCATCATC | GACGAACCCAAATTCAGCAT | 6-FAM | 1.5 |
| WFes0001770 | GTGCACACTGCCAACAGACT | AAGACTGCGGATTTGTTTCG | 6-FAM | 1.5* |
| WFes0001992 | CGTCGCTAGCAATCAAACTG | CAGCCTCAGTATGCTCCACA | 6-FAM | 3.5 |
| WFes0002404 | CGGTCTACCTGCTGGCTAAG | CGGTACTCCGTTTCTTTCCA | HEX | 3.5 |
| WFes0002465 | GATAGCGGTGGCTTGAGTTT | TTAGTCCGCTCCGGTCTATG | HEX | 3.5 |
| WFes0002528 | TTTACCTTGGCCTGGTTGAC | TAAAATGCAACATTCCATGC | 6-FAM | 1.5 |
| WFes0002563 | CTGGCAAAAAGAAGGGTGAA | ACGAAGCAGTTCGAAAGAGG | HEX | 1.5 |
| WFes0002696 | GCGATCCTGAATTGGACACT | GTGATCCATCAGACCCGACT | HEX | 1.5 |
| WFes0002931 | ACCTACGAGGCTGACGAGAA | CTCGGCATCGTGTTTGTATG | TET | 3.5 |
| WFes0002936 | TTCGGTGGCTTCTAAATTCG | GATTGATTCGTTCCGCATCT | TET | 1.5 |
| WFes0003015 | CAGATGCTGACCTCCTCTCC | TTCGGAAGTCATCACTAACTGG | HEX | 1.5 |
| WFes0003056 | ACAACCCCAATGTCAAATCC | GGGAAAAACTCAATCGCAAA | HEX | 1.5 |
| WFes0003178 | TCCGCATTACACCACTGCTA | TGCACAGCTTGAAAACGAAA | 6-FAM | 3.5* |
| WFes0003187 | CAGGCAACACCAACATCAAC | TTTCGTCATCATCGTTTGGA | HEX | 3.5 |
| WFes0003196 | CCGCTACCTGGTGATTTCAT | GTGTTACTGGGGGACGAGAA | 6-FAM | 1.5 |
| WFes0003617 | CACGAGGTCCACCACAAAAT | CCGGTTTAGGGCAAAGAATA | TET | 1.5 |
| WFes0003698 | GGAGTTACTGCCTCCCTTGA | TGAAGAGCCACTGCTGTTTG | HEX | 1.5* |
| WFes0004129 | GGGAGTTCCAAACAATCGAA | TGAGCCTCTATCGCACAAGA | 6-FAM | 1.5 |
| WFes0004208 | CGTACTCTGCACCCCAAAAT | TGGTCGAATGGTAGGTGTCA | HEX | 3.5* |
| WFes0004276 | CCACTTGCGAGTCTCCTTCT | TGCGCATTTGAACACTTCTC | 6-FAM | 1.5 |
| WFes0004447 | TGGCGTAAATTTTCGTTTGA | ATGATGCACACACCCCAAC | TET | 1.5 |
| WFes0004614 | GCTAGCTCTTCGGATGATGG | TCTATGTGGGATGGGTTGGT | HEX | 1.5 |
| WFes0004775 | ATCTCGAGCAGTGGATCCTG | GCCTAATTTGCGACATCCAT | TET | 1.5 |
| WFes0004827 | ACAAGTCAATTCTGCCCACA | CAAAAGCGAAAACGGAAGTC | 6-FAM | 1.5 |
| WFes0005005 | CACGTTCGATGTGTGAAAATG | GGCAACCCACACAACTCTTT | HEX | 1.5 |
| WFes0005186 | GGTGACGACCGATTCCTCTA | CGATTGGAACTTACGCGAAT | 6-FAM | 1.5 |
| WFes0005389 | AAACCCACCACCATGTCATT | AAACCCACGATGTAAAATATGTG | 6-FAM | 1.5 |
| WFes0005731 | ACCTGAAGGCTCACCTGGTA | AGGACCAGGAGGACCAGATT | TET | 1.5 |
| WFes0006166 | CTCGCTCGCAGACTCTCTTT | GCTCCATTTGCCCATCTAAA | HEX | 1.5 |
| WFes0006196 | TCGGCTGATTTGCATTTCGT | AAACGATCTGCCCTGAATTG | TET | 1.5 |
| WFes0006227 | AAATGGCCAACACTTTACCG | AATAGGGGGCTGATTTTGGT | TET | 1.5 |
| WFes0006277 | GACGTTCAGCCGTTAGTTCAG | CTATGAGGCTCCTGGTCAGC | TET | 1.5 |
| WFes0006310 | TGGAAACTCAGCAACCATGA | CCCCATCCATATCCTCTGTG | HEX | 1.5 |
| WFes0006418 | GAAATGGACTTTTGCGTGGT | AAGAGGCAAGCAACGGTAAA | 6-FAM | 1.5 |
| WFes0007000 | GTGGATGGATCTCGGTGAAA | TGTCTGCCATCTCCAGACAG | TET | 3.5 |
| WFes0007001 | AACACAAATGCACGTCCAGA | TCATGACCGACTTGTTGAGC | 6-FAM | 1.5 |
| WFes0007148 | TTGAAACGAGTGTTCCAGCA | AACGGAACATCGAAAGCAAC | HEX | 1.5 |
| WFes0007327 | TGTGCGTGTTGCCCTTATTA | CTGCTTCAACTTTGCCATCA | TET | 1.5 |
| WFes0007705 | GAGGAGTCTTCGTCGTCGTC | TCCGTCGTATAGTCCTTCTCTCA | TET | 1.5* |
| WFes0007834 | AGGAGGATTTCCGCAAGTTT | GTCGGGACAAAAGAGTCTCG | HEX | 1.5 |
| WFes0007867 | AGGCGAGGATGAAATCATTG | GCCAATTTTGATTCGACGTT | TET | 1.5 |
| WFes0008065 | TGCTAACGAGGGGTATAGCAA | CGCTTAGTCTCCTGGAATCG | TET | 1.5 |
| WFes0008210 | ACGCCGTTGATTGAGAGACT | CGGGAGAAATTCAGTCCAAA | HEX | 1.5 |
| WFes0008344 | GTGTGGATGCGTGAGTTTTG | CGCGTGCACCAACAATAATA | 6-FAM | 1.5 |
| WFes0008371 | AATGGCACGGTAAAATACGC | ACACCCGACTGACGATTTTC | TET | 1.5 |
| WFes0008397 | TTTCATGCCATAGCAAAACG | ACGAAGAATCGGTCAACACC | 6-FAM | 1.5 |
| WFes0008416 | AAATGCGTCGGAGAAATGTT | GCAGCCATCAGTGAAAGTGA | TET | 1.5 |
| WFes0008608 | CATTCATTCCGGGAAATCAG | GCTCTTCGGTCTCTTCTTCG | 6-FAM | 1.5 |
| WFes0008693 | AACAAAACAAACGCAAAAGTGA | GGAAAGTCTCGCAGCTGAAC | 6-FAM | 1.5 |
| WFes0008711 | AAAGGTCGCTGTCGTCACTT | AGACACGTCCGTTCGTCTG | TET | 1.5 |
| WFes0009083 | CATGCGCGTAAGAAGAACAA | CCCCTATGCGCCAATTACTA | 6-FAM | 1.5 |
| WFes0009235 | ATCGTTCCACTTTTGGTTGC | GACCTGAGGTTGAGCGTAGC | TET | 1.5 |
| WFes0009325 | GGCTGCCGAAAGTAATGAAA | GCTCTGTAAACGGTCGCTGT | 6-FAM | 1.5* |
| WFes0009357 | GGCAACGATAGACCACGAAT | GTCATCTACGAAGGCGAAGC | 6-FAM | 1.5 |
| WFes0009449 | CATCGACAACTGCTGCAAAT | GACAAAATGGAGCCTCAACC | 6-FAM | 1.5 |
| WFes0009477 | CAAGGGTAGCAGGATCTATTTG | CCGATGAGATGCAGAATGAA | HEX | 3.5* |
| WFes0009489 | TCAGCCAGTTTGTGGAACAG | GCCAAAGCGTAGTGCAGAAT | TET | 1.5 |
| WFes0009598 | AACTGGGGAGTTCCTCAACC | GTGAACTTCGGGTGTGTCCT | HEX | 1.5 |
| WFes0009604 | TATGAAATGACACGGCAACG | ACATAGCCAGGCGTGTTTTT | HEX | 1.5 |
| WFes0010456 | TATTTGGTCCGGCATTTCAT | TACGGGGACTCTGCTGATTT | TET | 1.5 |
| WFes0010572 | TTTTCTTCCGGACTCACGTT | AGCGTCGTTGTTGATTTTCC | 6-FAM | 1.5 |
| WFes0010752 | ACACATGCACCAGATGGAAA | GGATGCTGGACTGGTCATTT | TET | 1.5 |
| WFes0011039 | CGTTACACCACAGACGGTTG | TGAAAGCAGCATGAATCCAG | HEX | 3.5 |
| WFes0011309 | GATTTCATCAGTCGGGTCGT | AAACCGAGAAGTTCCCTCGT | HEX | 1.5 |
| WFes0011345 | CTCTCTCTTTCGTGCGTGTG | ATTCCAACTTGCCCTGATTG | HEX | 3.5 |
| WFes0011375 | TTCGCAAATTCGGTCTAAGC | GCGCCCCTAAGTCCTAAAAC | TET | 3.5 |
| WFes0011411 | ATATGAAGTGGCCTCCAACG | CCATTCATTCCAACCCAATC | 6-FAM | 1.5 |
| WFes0011675 | TGAGGATGGTCGTACGCTTT | CAACATGAAACCCAAACACG | TET | 3.5 |
| WFes0011784 | GGGCAACAGTACGGAGGTAA | ATCGGACTTGTAACGGCAAC | 6-FAM | 1.5 |
| WFes0011982 | CTTCGGCAGCGAAAACTTC | GATAGCCTTTGCCAAACACG | TET | 1.5 |
| WFes0012318 | GTCATCGTCAATCGAGGCTA | CGTTGTCCACCCAATTATCC | HEX | 1.5 |
