# Supplementary material for: Intragenic tandem repeats in Daphnia magna: structure, function and distribution
Source: BMC Res Notes. 2009 Oct 6;2:206. doi: 10.1186/1756-0500-2-206 (PMC2763877; doi:10.1186/1756-0500-2-206)
Supplement: Additional file 2 — Description of the 74 polymorphic VNTR loci. EST locus: locus containing the VNTR; Size: size of the repeated motif; Sequence: repeat consensus sequence. N: number of repeats, [the number of perfect repeatsm with exact consensus sequence, is shown in brackets]; A: number of alleles. *: total number of alleles in loci with more than one repeated motif. He: Gene diversity. [file 1756-0500-2-206-S2.DOC]

## Additional file 2 . Description of the 74 polymorphic VNTR loci.

EST locus: locus containing the VNTR; Size: size of the repeated motif; Sequence: repeat consensus sequence. N: number of repeats, [the number of perfect repeatsm with exact consensus sequence, is shown in brackets]; A: number of alleles. *: total number of alleles in loci with more than one repeated motif. He: Gene diversity.

| **EST locus** | **Size** | **Sequence** | **N** | **A** | **He** |
| --- | --- | --- | --- | --- | --- |
| WFes0001245 | 12 | GGTTACGGTGGC | 2 (2) | 2 | 0.28 |
| WFes0001508 | 2 | AG | 10 (5) | 2 | 0.20 |
| WFes0001526 | 3 | TTA | 5 (5) | 4 | 0.66 |
| WFes0001668 | 3 | CAA | 7 (7) | 2 | 0.05 |
| WFes0001770 | 15 | AAAAAAAAAAAAAAG | 2 (1) | 5 | 0.67 |
| WFes0001992 | 18 | GGAGGCTGTTGTTGAATG | 9 (7) | 8 | 0.74 |
| WFes0002404 | 16 | TCGCCATTATCCTCAG | 2 (1) | 5 | 0.70 |
| WFes0002465 | 39 | AGCTCCAGTTTACTCTGCACCGGCCTACGAGGCCCCAGC | 4 (1) | 6 | 0.45 |
| WFes0002528 | 3 | GGT | 5 (5) | 3 | 0.57 |
| WFes0002563 | 9 | GAAGAGGAA | 9 (3) | 5 | 0.64 |
| WFes0002696 | 3 | TCA | 22 (5) | 3 | 0.39 |
| WFes0002931 | 36 | GCCCGCAAAATCATTTGCCCTTCTTCTACCCAACTT | 2 (1) | 3 | 0.46 |
| WFes0002936 | 3 | TTC | 6 (5) | 3 | 0.51 |
| WFes0003015 | 3 | AAC | 9 (8) | 3 | 0.61 |
| WFes0003056 | 18 | CCGTAATCTCCGTAACCG | 6 (1) | 5 | 0.51 |
| WFes0003178 | 3 | TTC | 8 (6) | 4 | 0.63 |
| WFes0003187 | 15 | CTCAACAAGACGAGC | 3 (2) | 2 | 0.31 |
| WFes0003196 | 21 | GTCTCGAATTTTGGCGGTCCA | 2 (2) | 2 | 0.44 |
| WFes0003617 | 3 | GTT | 5 (5) | 8 | 0.82 |
| WFes0003698 | 45 | AACCTCCACCAATGGCTGGGTTAGACTGGCAACCTGCAAAGCTCG | 2 (1) | 3 | 0.36 |
| WFes0004129 | 2 | CA | 12 (12) | 4 | 0.62 |
| WFes0004208 | 3 | TGA | 9 (9) | 3 | 0.55 |
| WFes0004276 | 3 | ATT | 18 (11) | 9 | 0.81 |
| WFes0004447 | 2 | GT | 11 (11) | 4 | 0.77 |
| WFes0004614 | 2 | GT | 8 (8) | 2 | 0.69 |
| WFes0004775 | 3 | CAA | 6 (6) | 2 | 0.50 |
| WFes0004827 | 3 | CGT | 5 (5) | 3 | 0.59 |
| WFes0005005 | 2 | TG | 10 (10) | 5 | 0.77 |
| WFes0005186 | 3 | TTA | 6 (5) | 4 | 0.51 |
| WFes0005389 | 2 | GT | 8 (8) | 2 | 0.48 |
| WFes0005731 | 9 | TGGACCTCC | 3 (2) | 3 | 0.20 |
| WFes0006166 | 2 | CA | 12 (9) | 4* | 0.66 |
|  | 3 | TAT | 6 (6) | 4* | 0.66 |
| WFes0006196 | 2 | CA | 14 (14) | 6 | 0.72 |
| WFes0006227 | 12 | CCAATGCCTGTG | 4 (1) | 4 | 0.59 |
| WFes0006277 | 27 | GCTGTTGGGCCGGTGCTTGGTAAGCGG | 2 (2) | 4 | 0.69 |
| WFes0006310 | 12 | GGATACCACGGA | 2 (2) | 2 | 0.31 |
| WFes0006418 | 18 | TAAGCGGACTCTGCTGGG | 3 (1) | 3 | 0.50 |
| WFes0007000 | 3 | AAC | 6 (5) | 2 | 0.40 |
| WFes0007001 | 2 | CA | 7 (7) | 5 | 0.78 |
| WFes0007148 | 6 | GTTTTT | 7 (2) | 5 | 0.74 |
| WFes0007327 | 3 | CAA | 8 (6) | 3 | 0.53 |
| WFes0007705 | 39 | GAAGGTAAGGACGAAGAAGCTGATGTTTGCAAAGTATAG | 4 (2) | 3 | 0.43 |
| WFes0007834 | 2 | CA | 8 (8) | 5 | 0.86 |
| WFes0007867 | 2 | CA | 8 (8) | 2 | 0.44 |
| WFes0008065 | 21 | GCGATGTCAGGGAATTTTACT | 2 (2) | 2 | 0.28 |
| WFes0008210 | 2 | GT/GA | 20 (10) | 4 | 0.48 |
| WFes0008344 | 2 | CT | 8 (8) | 7 | 0.77 |
| WFes0008371 | 19 | TTTTTTTTTTTGTTTTTCC | 2 (1) | 8 | 0.82 |
| WFes0008397 | 2 | CA | 19 (13) | 7 | 0.73 |
| WFes0008416 | 2 | CA | 5 (5) | 2 | 0.49 |
| WFes0008608 | 3 | CAA | 16 (5) | 5 | 0.67 |
| WFes0008693 | 2 | CA | 12 (9) | 5 | 0.65 |
| WFes0008711 | 2 | GT | 12 (9) | 4 | 0.66 |
| WFes0009083 | 2 | CT | 10 (10) | 3 | 0.36 |
| WFes0009235 | 3 | TGG | 5 (5) | 2 | 0.49 |
| WFes0009325 | 27 | TCTTATTCCGCACCAGCAAAAGAAGCT | 2 (1) | 10 | 0.82 |
| WFes0009357 | 30 | AGCCTTGTATTCCGAAGCGGAGTAAGATGG | 4 (1) | 4 | 0.67 |
| WFes0009449 | 3 | GTT | 7 (6) | 3 | 0.53 |
| WFes0009477 | 3 | GCT | 8 (7) | 3 | 0.66 |
| WFes0009489 | 2 | TA | 7 (7) | 2 | 0.38 |
| WFes0009598 | 27 | CCAACCGGCACCTACACCCGTTTGGAG | 2 (1) | 4 | 0.42 |
| WFes0009604 | 12 | AAAAGAAAAGAC | 2 (2) | 2 | 0.10 |
| WFes0010456 | 5 | AAAAG | 5 (4) | 2 | 0.44 |
| WFes0010572 | 2 | TG | 16 (12) | 4 | 0.54 |
| WFes0010752 | 3 | GCT | 7 (7) | 4 | 0.70 |
| WFes0011039 | 6 | TATTTT | 7 (2) | 5 | 0.67 |
| WFes0011309 | 10 | AAAAAAGAAA | 3 (1) | 2 | 0.10 |
| WFes0011345 | 2 | TG | 17 (8) | 4 | 0.48 |
| WFes0011375 | 16 | AACAATAATAAATAAT | 2 (1) | 3 | 0.49 |
| WFes0011411 | 10 | CATTTCCTTT | 2 (2) | 4 | 0.33 |
| WFes0011675 | 17 | ATTTAAATACCAAAGTT | 2 (2) | 2 | 0.05 |
| WFes0011784 | 9 | GTGGATATG | 3 (2) | 4 | 0.33 |
| WFes0011982 | 2 | CA | 6 (6) | 2 | 0.30 |
| WFes0012318 | 21 | TTTGTTTTGTCGAAATTTGT | 2 (1) | 6 | 0.81 |

.
