# Supplementary material for: Intragenic tandem repeats in Daphnia magna: structure, function and distribution
Source: BMC Res Notes. 2009 Oct 6;2:206. doi: 10.1186/1756-0500-2-206 (PMC2763877; doi:10.1186/1756-0500-2-206)
Supplement: Additional file 3 — Results of homology searches and putative functions of EST loci. TM: transmembrane regions. Function categories: DEF: defense; MET: metabolism; OTH: other; SIG: signaling and gene expression regulation; SUR: surface and integumental proteins; UNK: unknown function. No hit: no significant homolog found (cut-off E-value 0.0001). [file 1756-0500-2-206-S3.DOC]

**Additional file 3.** Results of homology searches and putative functions of EST loci. TM: transmembrane regions. Function categories: DEF: defense; MET: metabolism; OTH: other; SIG: signaling and gene expression regulation; SUR: surface and integumental proteins; UNK: unknown function. No hit: no significant homolog found (cut-off E-value 0.0001).

| **ESTlocus** | **repeat position** | **TM** | **localisation** | **homolog** | **Domains** | **gene familiy** | **Annotation** | **functional category** |
| --- | --- | --- | --- | --- | --- | --- | --- | --- |
| WFes0001176 | coding (polyK) | 0 | intra | ABS19976.1 | [IPR002048 ; IPR011992](http://www.ebi.ac.uk/interpro/IEntry?ac=IPR002048) | [PTHR23049](https://panther.appliedbiosystems.com/panther/family.do?clsAccession=PTHR23049:SF4) | Myosin light chain | OTH |
| WFes0001211 | coding | 0 | extra | [Q17BL9](http://www.ebi.ac.uk/interpro/ISpy?ac=Q17BL9) | no hit | [IPR000618](http://www.ebi.ac.uk/interpro/IEntry?ac=IPR000618) | Cuticle protein | SUR |
| WFes0001245 | coding | 2 | surface | [A2YC96](http://www.ebi.ac.uk/interpro/ISpy?ac=A2YC96) | no hit | no hit | n/a | UNK |
| WFes0001334 | unknown | n/a | unknown | no hit | no hit | no hit | n/a | UNK |
| WFes0001411^a^ | coding (polyK) | 0 | intra | AAX62410.1 | [IPR000626](http://www.ebi.ac.uk/interpro/IEntry?ac=IPR000626) | [IPR006846](http://www.ebi.ac.uk/interpro/IEntry?ac=IPR006846) | Ribosomal protein | MET |
| WFes0001508 | unknown | 2 | surface | no hit | no hit | no hit | n/a | UNK |
| WFes0001526 | unknown | 1 | surface | no hit | no hit | no hit | n/a | UNK |
| WFes0001581 | 5'UTR | 0 | intra | [Q684I7](http://www.ebi.ac.uk/interpro/ISpy?ac=Q684I7) | no hit | [IPR004000](http://www.ebi.ac.uk/interpro/IEntry?ac=IPR004000) | Actin | OTH |
| WFes0001668^b^ | unknown | 1 | surface | no hit | no hit | no hit | n/a | UNK |
| WFes0001770 | unknown | n/a | unknown | no hit | no hit | no hit | n/a | UNK |
| WFes0001881 | unknown | 0 | unknown | no hit | no hit | no hit | n/a | UNK |
| WFes0001992 | Coding (polyASHSTT) | 0 | unknown | [Q7YZI0](http://www.ebi.ac.uk/interpro/ISpy?ac=Q7YZI0) | no hit | no hit | Lectin | DEF |
| WFes0002110 | coding | 0 | unknown | [A4FTP4](http://www.ebi.ac.uk/interpro/ISpy?ac=A4FTP4) | no hit | no hit | n/a | UNK |
| WFes0002307 | coding | 1 | surface | [Q7Q1B4](http://www.ebi.ac.uk/interpro/ISpy?ac=Q7Q1B4) | no hit | [IPR000618](http://www.ebi.ac.uk/interpro/IEntry?ac=IPR000618) | Cuticle protein | SUR |
| WFes0002404 | coding | 0 | intra | [Q17J02](http://www.ebi.ac.uk/interpro/ISpy?ac=Q17J02) | no hit | [IPR002042](http://www.ebi.ac.uk/interpro/IEntry?ac=IPR002042) | Uricase | MET |
| WFes0002465 | coding | 0 | extra | [Q86GL3](http://www.ebi.ac.uk/interpro/ISpy?ac=Q86GL3) | no hit | no hit | Cuticle protein | SUR |
| WFes0002528 | coding(polyH) | 2 | surface | no hit | no hit | no hit | n/a | UNK |
| WFes0002563 | coding (polyE) | 0 | extra | 6T2X9 | no hit | no hit | Troponin | OTH |
| WFes0002696 | coding (polyS) | 1 | surface | [Q173D9](http://www.expasy.org/uniprot/Q173D9) | no hit | IPR009786 | thyroid hormone-inducible hepatic protein | MET |
| WFes0002718 | coding(polyYI) | 2 | surface | no hit | no hit | no hit | n/a | UNK |
| WFes0002769 | coding | 0 | intra | [Q29I45](http://www.ebi.ac.uk/interpro/ISpy?ac=Q29I45) | no hit | [IPR001152](http://www.ebi.ac.uk/interpro/IEntry?ac=IPR001152) | Thymosin beta-4 | OTH |
| WFes0002771 | coding | 3 | surface | [P05790](http://www.ebi.ac.uk/interpro/ISpy?ac=P05790) | no hit | no hit | Fibroin heavy chain precursor | UNK |
| WFes0002931 | coding | 0 | extra | [Q9VYM0](http://www.ebi.ac.uk/interpro/ISpy?ac=Q9VYM0) | no hit | [IPR000618](http://www.ebi.ac.uk/interpro/IEntry?ac=IPR000618) | Cuticle protein | SUR |
| WFes0002936 | 3'UTR | 0 | intra | [P18091](http://www.expasy.org/uniprot/P18091) | IPR002048,; IPR011992 ; IPR014837 | [PTHR11915:SF47](https://panther.appliedbiosystems.com/panther/family.do?clsAccession=PTHR11915:SF47) | Alpha-actinin | OTH |
| WFes0003015 | 3'UTR | 0 | intra | ABS19976.1 | [IPR002048 ; IPR011992](http://www.ebi.ac.uk/interpro/IEntry?ac=IPR002048) | [PTHR23049:SF4](https://panther.appliedbiosystems.com/panther/family.do?clsAccession=PTHR23049:SF4) | Myosin light chain | OTH |
| WFes0003056 | coding | 0 | extra | [A0ESK0](http://www.ebi.ac.uk/interpro/ISpy?ac=A0ESK0) | no hit | no hit | Eggshell protein | SUR |
| WFes0003178 | unknown | 2 | surface | no hit | no hit | no hit | n/a | UNK |
| WFes0003186 | coding | 1 | surface | no hit | no hit | no hit | n/a | UNK |
| WFes0003187 | unknown | n/a | unknown | no hit | no hit | no hit | n/a | UNK |
| WFes0003196 | unknown | n/a | unknown | no hit | no hit | no hit | n/a | UNK |
| WFes0003448 | 3'UTR | 0 | intra | UPI000051547A | [IPR001781](http://www.ebi.ac.uk/interpro/IEntry?ac=IPR001781) | [PTHR18973:SF74](https://panther.appliedbiosystems.com/panther/family.do?clsAccession=PTHR18973:SF74) | Zinc finger LIM type | SIG |
| WFes0003510 | coding | 0 | extra | [A7L487](http://www.ebi.ac.uk/interpro/ISpy?ac=A7L487) | no hit | [IPR000618](http://www.ebi.ac.uk/interpro/IEntry?ac=IPR000618) | Cuticle protein | SUR |
| WFes0003617 | unknown | 0 | unknown | no hit | no hit | no hit | n/a | UNK |
| WFes0003650 | coding | 0 | intra | [A8JJH5](http://www.ebi.ac.uk/interpro/ISpy?ac=A8JJH5) | no hit | no hit | n/a | UNK |
| WFes0003698 | coding | 0 | intra | [Q8CH69](http://www.ebi.ac.uk/interpro/ISpy?ac=Q8CH69) | no hit | [IPR010304](http://www.ebi.ac.uk/interpro/IEntry?ac=IPR010304) | Survival motor neuron | MET |
| WFes0003828 | coding | 0 | extra | [Q17BL9](http://www.ebi.ac.uk/interpro/ISpy?ac=Q17BL9) | no hit | [IPR000618](http://www.ebi.ac.uk/interpro/IEntry?ac=IPR000618) | Cuticle protein | SUR |
| WFes0004100 | unknown | 2 | surface | no hit | no hit | no hit | n/a | UNK |
| WFes0004129 | unknown | 0 | unknown | no hit | no hit | no hit | n/a | UNK |
| WFes0004208 | unknown | 0 | unknown | no hit | no hit | no hit | n/a | UNK |
| WFes0004276 | 3'UTR | 0 | intra | Q7PMM3 | [IPR012677](http://www.ebi.ac.uk/interpro/IEntry?ac=IPR012677) | PTHR11546 | Nucleotide binding | SIG |
| WFes0004447 | unknown | 0 | unknown | no hit | no hit | no hit | n/a | UNK |
| WFes0004614 | unknown | 1 | surface | no hit | no hit | no hit | n/a | UNK |
| WFes0004775 | unknown | 0 | intra | no hit | no hit | no hit | n/a | UNK |
| WFes0004827 | unknown | 1 | surface | no hit | no hit | no hit | n/a | UNK |
| WFes0005005 | 5'UTR | 0 | intra | UPI0000D56A96 | no hit | PTHR16160 | n/a | UNK |
| WFes0005186 | unknown | 0 | extra | no hit | IPR000020; IPR001007 | no hit | Anaphylatoxin | DEF |
| WFes0005389 | unknown | 2 | surface | no hit | no hit | no hit | n/a | UNK |
| WFes0005731 | coding (poly DLL) | 0 | intra | P88822 | [IPR000005](http://www.ebi.ac.uk/interpro/IEntry?ac=IPR000005) | [PTHR10499](https://panther.appliedbiosystems.com/panther/family.do?clsAccession=PTHR10499) | Helix-turn-helix protein | SIG |
| WFes0006166 | unknown | 0 | unknown | no hit | no hit | no hit | n/a | UNK |
| WFes0006196 | 5'UTR | 1 | surface | no hit | no hit | no hit | n/a | UNK |
| WFes0006227 | coding (polyMPVP) | 0 | intra | [Q7TN25](http://www.ebi.ac.uk/interpro/ISpy?ac=Q7TN25) | no hit | [IPR001079](http://www.ebi.ac.uk/interpro/IEntry?ac=IPR001079) | Galectin | DEF |
| WFes0006277 | coding | 0 | unknown | [A8X4B5](http://www.ebi.ac.uk/interpro/ISpy?ac=A8X4B5) | no hit | no hit | n/a | UNK |
| WFes0006310 | coding | 0 | unknown | [A0P8W6](http://www.ebi.ac.uk/interpro/ISpy?ac=A0P8W6) | no hit | no hit | n/a | UNK |
| WFes0006418 | coding | 0 | extra | [A7L487](http://www.ebi.ac.uk/interpro/ISpy?ac=A7L487) | no hit | [IPR000618](http://www.ebi.ac.uk/interpro/IEntry?ac=IPR000618) | Cuticle protein | SUR |
| WFes0007000 | unknown | 1 | surface | no hit | no hit | no hit | n/a | UNK |
| WFes0007001 | unknown | 0 | unknown | no hit | no hit | no hit | n/a | UNK |
| WFes0007148 | unknown | 0 | unknown | no hit | no hit | no hit | n/a | UNK |
| WFes0007327 | unknown | 0 | unknown | no hit | no hit | no hit | n/a | UNK |
| WFes0007705 | coding | 0 | intra | [A9REA5](http://www.ebi.ac.uk/interpro/ISpy?ac=A9REA5) | no hit | no hit | n/a | UNK |
| WFes0007779^c^ | coding (polyK) | 0 | intra | UPI00005A0EF0 | no hit | IPR003578; IPR013753 | GTP binding protein | SIG |
| WFes0007834 | 3'UTR | 1 | surface | UPI000155C417 | no hit | no hit | n/a | UNK |
| WFes0007867 | 5'UTR | 1 | surface | UPI0000615EF2 | [IPR003598 ; IPR007110; IPR013098; IPR013783](http://www.ebi.ac.uk/interpro/IEntry?ac=IPR003598) | PTHR10489:SF39 | Immunoglobulin-like | SIG |
| WFes0007965 | unknown | 0 | unknown | no hit | no hit | no hit | n/a | UNK |
| WFes0008065 | unknown | n/a | unknown | no hit | no hit | no hit | n/a | UNK |
| WFes0008210 | 5'UTR | 0 | intra | [Q7KVL6](http://www.ebi.ac.uk/interpro/ISpy?ac=Q7KVL6) | no hit | no hit | Actin organization | OTH |
| WFes0008344 | unknown | 0 | unknown | no hit | no hit | no hit | n/a | UNK |
| WFes0008371 | unknown | n/a | unknown | no hit | no hit | no hit | n/a | UNK |
| WFes0008397 | unknown | 0 | unknown | no hit | no hit | no hit | n/a | UNK |
| WFes0008416 | unknown | 0 | unknown | no hit | no hit | no hit | n/a | UNK |
| WFes0008608 | coding(polyQ) | 1 | surface | [A7RLU4](http://www.ebi.ac.uk/interpro/ISpy?ac=A7RLU4) | no hit | no hit | n/a | UNK |
| WFes0008693 | unknown | 0 | unknown | no hit | no hit | no hit | n/a | UNK |
| WFes0008711 | unknown | 0 | unknown | no hit | no hit | no hit | n/a | UNK |
| WFes0008717 | coding (polyP) | 0 | intra | [Q16K93](http://www.ebi.ac.uk/interpro/ISpy?ac=Q16K93) | [IPR013992](http://www.ebi.ac.uk/interpro/IEntry?ac=IPR013992) | [IPR001837](http://www.ebi.ac.uk/interpro/IEntry?ac=IPR001837) | Cyclase associated protein | SIG |
| WFes0008857 | 5'UTR | 0 | intra | [Q7RTS9](http://www.ebi.ac.uk/interpro/ISpy?ac=Q7RTS9) | no hit | [PTHR12895](https://panther.appliedbiosystems.com/panther/family.do?clsAccession=PTHR12895) | Dymeclin | UNK |
| WFes0008922 | unknown | 0 | unknown | no hit | no hit | no hit | n/a | UNK |
| WFes0009056 | coding | 1 | surface | Q4CPM9 | IPR008262 | no hit | Lipase | MET |
| WFes0009083 | unknown | 0 | unknown | no hit | no hit | no hit | n/a | UNK |
| WFes0009164 | coding | 0 | intra | Q7QB17 | IPR014038; IPR001326; IPR014717 | PTHR11595 | Translatio eleongation factor | MET |
| WFes0009235^d^ | Coding (polyG) | 0 | extra | P20621 | IPR08160 | PTHR10499 | Cuticle protein | SUR |
| WFes0009325 | coding | 0 | unknown | A818T3 | No hit | No hit | SNF2 superfamily protein | UNK |
| WFes0009357 | coding | 0 | extra | Q86GK9 | No hit | No hit | Cuticle protein | SUR |
| WFes0009449 | Coding (PolyN) | 1 | surface | Q9U9S7 | No hit | No hit | Adenylyl cyclase | MET |
| WFes0009477 | Coding (polyQ) | 0 | intra | A2A776 | IPR015880 | IPR002627 | tRNA isopentenyltransferase | MET |
| WFes009489 | 3’UTR | 1 | surface | Q6V4H5 | IPR001238; IPR002350; IPR011497 | PTHR21312 | Serine proteinase inhibitor | MET |
| WFes009598 | coding | 0 | extra | Q7QEL5 | No hit | No hit | Cuticle protein | SUR |
| WFes0009604 | unknown | n/a | No hit | No hit | No hit | No hit | n/a | UNK |
| WFes0009709^e^ | coding | 0 | extra | A0N015 | PF00379; PS51155; PS00233 | PTHR10380 | Cuticle protein | SUR |
| WFes0009967 | unknown | 0 | intra | Q8C2N7 | No hit | PTHR18973 | LIM domain containing protein | OTH |
| WFes0010127^f^ | Coding (polyE) | 0 | intra | Q7ZY81 | No hit | IPR002164 | Nucleosome assembly protein | SIG |
| WFes0010139 | unknown | 0 | unknown | No hit | No hit | No hit | n/a | UNK |
| WFes0010163 | 3’UTR | 2 | surface | A9POR6 | No hit | IPR007667 | Hypoxia induced protein | DEF |
| WFes0010456^g^ | unknown | 0 | unknown | No hit | No hit | No hit | n/a | UNK |
| WFes0010572 | unknown | 0 | unknown | Ho hit | No hit | No hit | n/a | UNK |
| WFes0010752 | Coding (polyA) | 1 | surface | A0J1Q9 | No hit | No hit | n/a | UNK |
| WFes0011039^h^ | 3’UTR | 0 | intra | Q9XZT5 | No hit | IPR001023 | Heat-shock protein | DEF |
| WFes0011235 | unknown | n/a | unknown | No hit | No hit | No hit | n/a | UNK |
| WFes0011309 | unknown | n/a | unknown | No hit | No hit | No hit | n/a | UNK |
| WFes0011345 | unknown | 0 | unknown | No hit | No hit | No hit | n/a | UNK |
| WFes0011375 | 5’UTR | 0 | intra | Q5TMY7 | IPR001251 | IPR001071 | Cellular retinaldehyde binding | SIG |
| WFes0011411^i^ | unknown | n/a | unknown | No hit | No hit | No hit | n/a | UNK |
| WFes0011622 | Coding (polyK) | 0 | intra | Q0ZB78 | IPR002735 | PD004078; PTHR23001 | Translation initiation factor | MET |
| WFes0011675 | Coding/3’UTR | 0 | intra | Q2KKX3 | No hit | IPR000626 | Ubiquitin | SIG |
| WFes0011784 | Coding (diTIS) | 2 | surface | A0ESK0 | No hit | No hit | Eggshell protein | SUR |
| WFes0011918^j^ | 3’UTR | 0 | intra | Q7QHP7 | No hit | IPR007122; PTHR11977 | Gelsolin | OTH |
| WFes0011982 | 5’UTR | 0 | intra | Q6PPH8 | No hit | IPR001925; PTHR11743 | Porin | OTH |
| WFes0012318 | unknown | n/a | unknown | No hit | No hit | No hit | n/a | UNK |

a : *D. pulex* homologue: scaffold 70, position 606869-607000

b: *D. pulex* homologue: scaffold 2, position 2475539-2475405

c: *D. pulex* homologue: scaffold 152, position 83238-83532

d: *D. pulex* homologue: scaffold 47, position 179195-178922

e : *D.pulex* homologue : scaffold 37, position 501283-501639

f : *D. pulex* homologue: scaffold 1, position 3975751-3975386

g : *D. pulex* homologue: scaffold 675, position 19174-18675

h : *D. pulex* homologue: scaffold 145, position 140644-140981

i : *D. pulex* homologue: scaffold 15, position 499046-499291

j : *D. pulex* homologue: scaffold 1, position 913197-912784
