# Supplementary material for: Intragenic tandem repeats in Daphnia magna: structure, function and distribution
Source: BMC Res Notes. 2009 Oct 6;2:206. doi: 10.1186/1756-0500-2-206 (PMC2763877; doi:10.1186/1756-0500-2-206)
Supplement: Additional file 4 — Distribution of allele sizes of polymorphic loci in each sampled location. [file 1756-0500-2-206-S4.DOC]

**Additional file 4**. Distribution of allele sizes of polymorphic loci in each sampled location.

|  | Location | | | | | |
| --- | --- | --- | --- | --- | --- | --- |
| Locus | Germany | Belgium | Finland | Hungary | UK | Canada |
| WFes0001245 | 353 | 353 | 353 | 353 | 353 | 346 |
| WFes0001508 | 162 | 154  162 | 162 | 162 | 154  162 | 161 |
| WFes0001526 | 213 | 210 | 210  213 | 208  210  213 | 213 | 205 |
| WFes0001668 | 273 | 273 | 273 | 273 | 273 | 266  273 |
| WFes0001770 | 385  412 | 385  388 | 382  385  390 | 382  385  388 | 382 | 382 |
| WFes0001992 | 437  532 | 399  413  472 | 437  491 | 399  437  472 | 454  510 | 437 |
| WFes0002404 | 244 | 244  268  274 | 244 | 231  244  268 | 274 | 167  231 |
| WFes0002465 | 217  255 | 217 | 217 | 182  217  235  255 | 163  217 | 157  217 |
| WFes0002528 | 314  316  318 | 314  316  318 | 314  316 | 316  318 | 314 | 314 |
| WFes0002563 | 253  256  268 | 253  256  262 | 256 | 238  253  256 | 253 | 253 |
| WFes0002696 | 365  368 | 365 | 365 | 365  368  371 | 365  368 | 365 |
| WFes0002931 | 320 | 320 | 354 | 320  354 | 320  340 | 320 |
| WFes0002936 | 265 | 265  268 | 265 | 265 | 263 | 263 |
| WFes0003015 | 313  315 | 313  315  317 | 315 | 313  315  317 | 313 | 313 |
| WFes0003056 | 387 | 387  399 | 387 | 384  387  402 | 384  387 | 347 |
| WFes0003178 | 254 | 251  254  257 | 254 | 254 | 257 | 248 |
| WFes0003187 | 391 | 379  391 | 391 | 379  391 | 379  391 | 391 |
| WFes0003196 | 273 | 252  273 | 273 | 273 | 252  273 | 252 |
| WFes0003617 | 245  249  250  252 | 243  245  247  250 | 242  243  252 | 244  247  250 | 245  247  249 | 249 |
| WFes0003698 | 380  414 | 380 | 380 | 380 | 380 | 338 |
| WFes0004129 | 237  239 | 230  237  239 | 239 | 234  237 | 234  239 | 239 |
| WFes0004208 | 363  368 | 363  368 | 363  368 | 363  366  368 | 363 | 368 |
| WFes0004276 | 298  304 | 290  294 | 298 | 304  307 | 287  290 | 279 |
| WFes0004447 | 197  200 | 197  200  204 | 202 | 202 | 204 | 200 |
| WFes0004614 | 457 | 457 | 453  455 | 455  457  460 | 453 | 455 |
| WFes0004775 | 221  223 | 221  223 | 221  223 | 223 | 223 | 221 |
| WFes0004827 | 138 | 138  139 | 139 | 139 | 139 | 137  138 |
| WFes0005005 | 121  126 | 119  121  128 | 121 | 119  121  126 | 119  124 | 126  128 |
| WFes0005186 | 403  413 | 403 | 403 | 403  409 | 403 | 409 |
| WFes0005389 | 176  180 | 176  180 | 176 | 176  180 | 176 | 180 |
| WFes0005731 | 217 | 217 | 217 | 217  224 | 213  217 | 217 |
| WFes0006166 | 168  171  173 | 171  173 | 164  173 | 168  171 | 173 | 171  173 |
| WFes0006196 | 368  372  386 | 372 | 368  376 | 366  368  376  380 | 368 | 380 |
| WFes0006227 | 403  414 | 414 | 414 | 403  414  439 | 403  411 | 411  414 |
| WFes0006277 | 194  222  247 | 194  220  222  247 | 194  220 | 194  222 | 220  222 | 194 |
| WFes0006310 | 325 | 313  325 | 325 | 325 | 313  325 | 313  325 |
| WFes0006418 | 331 | 331  361 | 331 | 331  361 | 331 | 329 |
| WFes0007000 | 231 | 231  273 | 273 | 231  273 | 231 | 231 |
| WFes0007001 | 371 | 368  371  373  375 | 366 | 366  375 | 371 | 361 |
| WFes0007148 | 236  242 | 242  251 | 242 | 236  242  251 | 243 | 248 |
| WFes0007327 | 328  331 | 328  331 | 328 | 328  331 | 331 | 331 |
| WFes0007705 | 336  342 | 336 | 336 | 295  342 | 336 | 336 |
| WFes0007834 | 330  332 | 330  332 | 332  334 | 330  334 | 330  334 | 336  341 |
| WFes0007867 | 340 | 342 | 340 | 340 | 342 | 340 |
| WFes0008065 | 366 | 366 | 366 | 366 | 366 | 346 |
| WFes0008210 | 217  219 | 217  219 | 219 | 217  219 | 219 | 219 |
| WFes0008344 | 116  120  122 | 116  122  124  126 | 116  120 | 116  118  122  124  126  130 | 120  122 | 120 |
| WFes0008371 | 186  187 | 186  188 | 186  189 | 188  192  205 | 187  188 | 196  197 |
| WFes0008397 | 262  272 | 262 | 262  266 | 250  262  264  266 | 258  262 | 253 |
| WFes0008416 | 393  397 | 393 | 397 | 397 | 393 | 397 |
| WFes0008608 | 292 | 292  294 | 294 | 292  298  302 | 292  294 | 290 |
| WFes0008693 | 297 | 297  302  310 | 297  314 | 297  314 | 297  314 | 302  304 |
| WFes0008711 | 177  179 | 173  177  179 | 175  177 | 173  175  177 | 177 | 179 |
| WFes0009083 | 366 | 366 | 366 | 359  363  366 | 363 | 366 |
| WFes0009235 | 311 | 311  314 | 314 | 311 | 314 | 314 |
| WFes0009325 | 336  343  359 | 351  376  379  413 | 386 | 334  345  376  379 | 386 | 334  343 |
| WFes0009357 | 413 | 413  467 | 381  383  411 | 411  413 | 383  413 | 383 |
| WFes0009449 | 195 | 195  198 | 195  198 | 195  198 | 195 | 208 |
| WFes0009477 | 319  322  325 | 319  325 | 319  322 | 319  325 | 319 | 322 |
| WFes0009489 | 318 | 315  318 | 318 | 315  318 | 315 | 318 |
| WFes0009598 | 384  386  412 | 384  386 | 381  386 | 386 | 386 | 386 |
| WFes0009604 | 203 | 203  214 | 203  214 | 203 | 203 | 203 |
| WFes0010456 | 452  455 | 452  455 | 452 | 452  455 | 455 | 455 |
| WFes0010572 | 146 | 139  143  146 | 139  151 | 143  146 | 146 | 146 |
| WFes0010752 | 265  271 | 265  268  271  274 | 268  271 | 265  271  274 | 268 | 271 |
| WFes0011039 | 349  359 | 344  349 | 344  349 | 278  349 | 344 | 346 |
| WFes0011309 | 126 | 126 | 126 | 126 | 126 | 126  134 |
| WFes0011345 | 227  233 | 225  233 | 233 | 227  233 | 233 | 231 |
| WFes0011375 | 381  410 | 381  410  415 | 381 | 381 | 415 | 381 |
| WFes0011411 | 254 | 248  254 | 254 | 254 | 254 | 206  268 |
| WFes0011675 | 201 | 197  201 | 201 | 201 | 201 | 201 |
| WFes0011784 | 282 | 282 | 282 | 273  282 | 282 | 276  285 |
| WFes0011982 | 482 | 482 | 482 | 482 | 482 | 484 |
| WFes0012318 | 235  237  244 | 244 | 233 | 237  242  244 | 235 | 246 |
